# Privacy-Preserving Deep Learning for the Detection of Protected Health Information in Real-World Data: Comparative Evaluation

**DOI:** 10.2196/14064

**Published:** 2020-05-05

**Authors:** Sven Festag, Cord Spreckelsen

**Affiliations:** 1 Department of Medical Informatics Medical Faculty RWTH Aachen University Aachen Germany; 2 Institute of Medical Statistics, Computer and Data Sciences Jena University Hospital Jena Germany

**Keywords:** privacy-preserving protocols, neural networks, health informatics, distributed machine learning

## Abstract

**Background:**

Collaborative privacy-preserving training methods allow for the integration of locally stored private data sets into machine learning approaches while ensuring confidentiality and nondisclosure.

**Objective:**

In this work we assess the performance of a state-of-the-art neural network approach for the detection of protected health information in texts trained in a collaborative privacy-preserving way.

**Methods:**

The training adopts distributed selective stochastic gradient descent (ie, it works by exchanging local learning results achieved on private data sets). Five networks were trained on separated real-world clinical data sets by using the privacy-protecting protocol. In total, the data sets contain 1304 real longitudinal patient records for 296 patients.

**Results:**

These networks reached a mean F1 value of 0.955. The gold standard centralized training that is based on the union of all sets and does not take data security into consideration reaches a final value of 0.962.

**Conclusions:**

Using real-world clinical data, our study shows that detection of protected health information can be secured by collaborative privacy-preserving training. In general, the approach shows the feasibility of deep learning on distributed and confidential clinical data while ensuring data protection.

## Introduction

### Background

Data protection is a major issue in health care, but clinical research and medical care also rely on high accessibility of patient data. The problem is aggravated in the context of data-driven medicine, where the amount of data needed exceeds the capacity of manual data curation and manual deidentification that would be necessary to protect patient privacy. In the United States, the Health Insurance Portability and Accountability Act of 1996 obliges data curators to remove protected health information (PHI) from medical records before they are shared with researchers. The same holds true for many other countries (see, for instance, §6 GDSG NW [Germany], SI 1438/2002 reg. 7 [England and Wales]). Computer-based data deidentification primarily needs to solve the task of detecting personal information like names, phone numbers, locations, etc. Deidentification systems must meet two opposed interests. To ensure privacy, they must work with high sensitivity (ie, avoid overlooking personal data). Additionally, these systems need to maintain high specificity (ie, avoid removing data unnecessarily). Otherwise, deidentified texts would contain little valuable information compared with the original inputs.

Many approaches to finding protected personal information in health records are based on supervised machine learning [1-3]. Such systems have proven to be very efficient for the deidentification task. One presented by Dernoncourt et al [3] even outperformed other state-of-the-art approaches in 2016. The general shortcoming of such approaches in this context is that they depend heavily on labeled training data. These training data usually consist of original health records containing personal information. Thus, these data cannot be shared among researchers for distributed training due to the above-mentioned problem. Consequently, there are many small training data sets at medical research institutes which can only be used locally. An additional challenge arises from the fact that even trained neural networks (NNs) can be abused to recover PHI that has been used for training [4,5]. Hence, a trained network for deidentification cannot be shared with other researchers or the public without causing a threat to patient privacy.

### Related Work

Several systems for the deidentification of PHI have been presented in the last 20 years. Meystre et al [6] state that most of the systems introduced before 2010 are rule-based or rely on pattern matching. The underlying patterns and rules are typically hand-crafted. Thus, domain experts are needed for the costly generation of such systems resulting in limited generalizability [6]. More recent approaches are mainly based on machine learning or, more precisely, conditional random fields and support vector machines [7]. For these systems, only features of the input data must be specified by hand. Several such tools, however, use additional rules for identifying certain classes of PHI or postprocessing [1,2,8]. The method proposed by Dernoncourt et al [3] is one of the first that solely makes use of NNs and thus is independent of manual feature selection. Liu et al [9] compare several rule-based and NN-based approaches to the deidentification task. Moreover, they introduce an ensemble method that combines these systems.

Collaborative privacy-preserving machine learning has already been studied by several authors. Many systems introduced in this field homomorphically encrypt local data and share the ciphered information for centralized training [10-12]. Thus, these systems need an authority trusted by all parties to start encrypted communication. Another issue of cryptographic centralized training is the cost for encryption and decryption which can become untenable for high-dimensional data in practical applications [10]. By using a decentralized training which obviates the need for sharing individually identifiable health information, these problems vanish. Chang et al [13] experimented with several nonparallel distributed training heuristics. In their proposed framework, local workers conceal their training data but share full sets of learned network parameters. According to Shokri and Shmatikov [4], this transfer of local parameters might still lead to indirect leakage of personal information. Moreover, Fredrikson et al [5] investigated model inversion attacks that infer sensitive information included in the training data from a given trained model. The authors distinguish between black- versus white-box attacks: black-box attacks exploit prediction/classification queries via functional access to a model without knowing its internal details, while white-box attacks additionally use details of the model (eg, network topology and weight matrices). Fredrikson et al [5] demonstrated the feasibility of model inversion attacks especially in the case of white-box approaches using real-world data and publicly available trained classifiers. Their experiments included NNs used for facial recognition. A model inversion attack was able to reconstruct faces of the training set after entering the corresponding names, which can well be considered a relevant violation of privacy.

Hence, we decided to use the collaborative and parallel training method introduced by Shokri and Shmatikov [4]. It reduces the risk of indirect leakage and allows the cooperation of institutes with different computational capacities. For this training scheme, no encryption or any other data protection is needed except the local data and computations must be secured. Like most other collaborative privacy-preserving learning approaches, it only protects from honest-but-curious adversaries. That means malicious participants can hamper the learning or even render it unusable, but they cannot gain information not meant for them. A similar training approach was used by Liu et al [14]. In contrast to our study, they trained feedforward NNs and investigated the effect of using mobile devices to conduct the local training.

### Aim of the Study

Our study is aimed at showing the feasibility of a collaborative privacy-preserving learning approach for deep NNs trained to detect personal information in real-world clinical data. We adopted the network topology of the mentioned deidentification approach described by Dernoncourt et al [3]. To overcome the problem of small data sets and privacy issues, we used collaborative privacy-preserving learning suggested by Shokri and Shmatikov [4]. This technique allows the integration of many local data sets into the training while minimizing the risk of leaking protected information. It restricts the training to the local site of each data provider (ie, own data stay with each provider) and only relies on the sharing of small fractions of the local improvements.

We evaluated the performance of the deidentification network trained in a collaborative and privacy-preserving manner. For this purpose, we used an existing set of longitudinal clinical narratives published in the course of the Informatics for Integrating Biology and the Bedside (i2b2) 2014 deidentification challenge hosted by the National Center for Biomedical Computing [7,15] (deidentified clinical records used in this research were provided by the i2b2 National Center for Biomedical Computing funded by U54LM008748 and were originally prepared for the Shared Tasks for Challenges in Natural Language Processing for Clinical Data organized by Dr. Özlem Uzuner, i2b2, and the State University of New York).

## Methods

### Recurrent Neural Network for Deidentification

#### General Topology and Training Goal

The recurrent neural network (RNN) presented by Dernoncourt et al [3] is constructed to work on fragments of medical notes. The computed output is a sequence of labels with each label corresponding to one term (also called a token) of the input text. In our adapted version, a label can either be non-PHI or one of 28 PHI classes or subclasses adopted from the definitions of the i2b2 deidentification challenge (Table 1). If there is at least one subclass, the general class is not used as a label.

**Figure 1:**
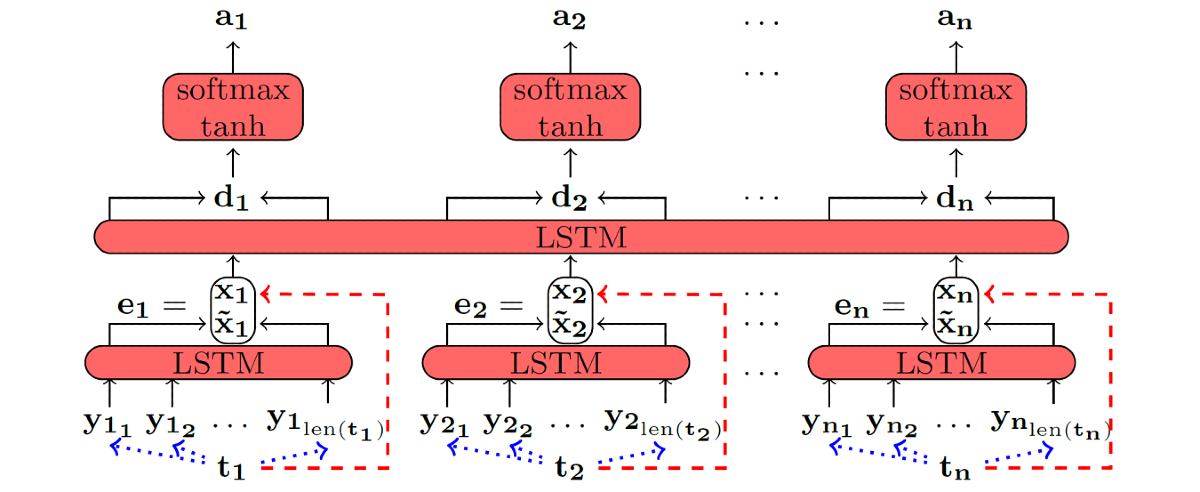
Network topology of the recurrent neural network used for deidentification [3].

The network topology shows two different layers. The first layer consists of long short-term memory (LSTM) RNNs and is called the character-enhanced token embedding layer. The actual label assignment is conducted in the label prediction layer seen in Figure 1.

**Table 1:** Possible protected health information classes of terms.

Class	Subclasses
Name	Patient, clinician, username
Profession	—
Location	Hospital, organization, street, city, state, country, zip, other
Age	—
Date	—
Contact	Phone, fax, email, URL, Internet Protocol address^a^
Identification	Social security number^a^, medical record number, health plan number, account number^a^, license number^a^, vehicle identification^a^, device identification, biometric identification, identification number

^a^Classes that are not present in the published data set [7].

#### Preprocessing

During preprocessing, the input text is subdivided into terms **t_i_** which are then transformed into their vector representations **x_i_** ∈ 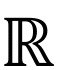
^300^. For the transformation we use the Word2Vec approach which can map words into a low-dimensional vector space while keeping some semantic and syntactic information [16]. This transformation from word to vector is depicted by the red-dashed arrows (Figure 1). As a result of the preprocessing, the input text *T* = (**t_1_,** ... **, t_n_**) is available as a sequence of Word2Vec vectors *X* = (**x_1_,** ... **, x_n_**).

In the second preprocessing step, every term **t_i_** is further divided into its individual characters (t_i_1_, ... , t_i_len(ti )_). Again, every character is translated into a vector **y_i_j_** ∈ {0,1}^128^ of low dimensionality. The translation is represented by blue-dotted arrows (Figure 1). In contrast to the word embedding, characters are encoded by a simple one-hot method (ie, by vectors with exactly one nonzero component). The dimension being nonzero is obtained by taking the Unicode representation of the corresponding character if it is smaller than 127. For all other characters, the last dimension is set to 1. In this way, all ASCII punctuations and symbols as well as the basic Latin alphabet can be encoded. All letters are mapped to corresponding one-hot vectors without taking any syntactic information into account.

To sum up, at the end of the preprocessing there are vectors **x_i_** (word embeddings) representing every input term and vectors **y_i_j_** (character embeddings) each representing one occurrence of a character.

#### Character-Enhanced Token Embedding Layer

For every word, the first layer contains an independent instance of a bidirectional LSTM. Hence, the number of RNNs changes for every input text.

LSTMs integrate new parts of an input sequence into their computation in every step. Furthermore, they keep some information of the already processed prefix of the sequence. This makes them capable of learning long-term dependencies between vectors whose positions in the input are far apart [17]. Mathematically, a general unidirectional LSTM can be described as follows.

Let *W_i_*, *W_c_*, *W_o_* be three weight matrices and **b_i_**, **b_c_**, **b_o_** be three bias vectors. These parameters are learned during the training. If the input sequence equals (z_1_, ... , z_m_), the LSTM conducts *m* steps. In the **t**^th^ iteration, the hidden state **h_t_** and the memory state **c_t_** are computed using **z_t_**, **h_t-1_**_,_ and **c_t-1_** as inputs and the following formulas:

**i_t_** = σ(*W_i_* · concat(**z_t_**, **h_t-1_**) + **b_i_** + **1**)

**c_t_** = **i_t_** ⊙ **c_t-1_** + (1 – **i_t_**) ⊙ tanh(*W_c_* · concat(**z_t_**, **h_t-1_**) + **b_c_**)

**o_t_** = σ(*W_o_* ⋅ concat(**z_t_**, **h_t-1_**) + **b_o_**)

**h_t_** = **o_t_** ⊙ tanh(**c_t_**)

where ⊙ denotes the element-wise multiplication, σ the element-wise logistic sigmoid function, and tanh the element-wise hyperbolic tangent function. For the first iteration the hidden state **h_0_** and the cell state **c_0_** must be initialized arbitrarily. Sometimes the full sequence of hidden states (**h_1_**, ... , **h_m_**) is considered the output of the LSTM and sometimes just the last hidden state **h_m_** is seen as the output.

A bidirectional LSTM consists of two independent unidirectional LSTMs. The first one works on the original input sequence (**z_1_**, ... , **z_m_**), whereas the second one computes the output for the reversed sequence (**z_m_**, … , **z_1_**).

By combining the two output sequences (**h_1_^for^**, ... , **hm^for^**) and (**h_1_^back^**, ... , **hm^back^**) one gets the overall output (concat(**h_1_^for^**, ... , **h_1_^back^**),..., concat(**hm^for^**, ... , **hm^back^**)) or concat(**h_m_^for^**, ... , **h_m_^back^**), respectively.

The token embedding layer contains one bidirectional LSTM per term **t_i_** of the input text. Every such LSTM works on the corresponding sequence of character embeddings (**y_i_1_**,...,**y_i_len(ti)_**). At this stage no information are exchanged between LSTMs working on different tokens. However, they all share the same weight matrices and bias vectors. The output of the **i**^th^ RNN can be written as 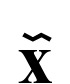
**_i_** =concat(**h_i_len(t_i)_^for^**, **h_i_len(t_i)_^back^**).

This vector is used as an additional word embedding to overcome shortcomings of Word2Vec [3]. The presented computation always leads to a reproducible vector representation of a token, whereas Word2Vec cannot handle vocabulary that was not used during training. Moreover, this strategy keeps more semantic information than pure Word2Vec combined with a preceding lemmatization step. However, in comparison to the Word2Vec embedding, those LSTMs compute vectors that do not contain any information of interword dependencies.

To keep the advantages of both strategies, the two word embeddings of every token are combined in one vector **e_i_** = concat(**x_i_**, 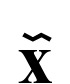
**_i_**). In summary, it can be stated that for the input sequence (**t_1_**, ... , **t_n_**) the character-enhanced token embedding layer computes the output (**e_1_**, ... , **e_n_**).

#### Label Prediction Layer

The subsequent layer evaluates the dependencies between different words of the input text. Again, a bidirectional LSTM is used for the task. In contrast to the previous layer, only one LSTM is used independent of the number of tokens. This RNN uses the output sequence (**e_1_**, ... , **e_n_**) of the previous layer as its input. The output consists of the full sequence (**d_1_**, ... , **d_n_**) where **d_i_** = concat(**h_1_^for^**, ... , **h_1_^back^**).

Every **d_i_** is further processed by a two-layer, fully connected feedforward network. The parameters are the same for every input **d_i_** and are trained jointly for every sequence (**d_1_**, ... , **d_n_**). The network works as follows:

**l_i_** = tanh(*W_1_* · **d_1_** + **b_1_**) hidden layer

**a_i_** = softmax(*W_2_* · **l_i_** + **b_2_**) output layer

Thus, the results **a_i_** ∈ [0,1]^29^ can be interpreted as conditional posterior probabilities of the possible labels given the input token **t_i_**. By choosing label(**t_i_**) = argmax_j∈{1,...,29}_ **a_i_**__j,_ every word is uniquely assigned one of the 28 PHI classes (Table 1) or the non-PHI label. The loss is defined pursuant to the cross-entropy and minimized during training.

### Collaborative Training

#### Aim

The aim of collaborative learning is the integration of many (private) training sets into the learning process. Note that this goal is different from the one of distributed training that solely aims at faster training through the use of several computation nodes. The following sections summarize three alternative collaborative approaches: the nonprotective standard method, a round robin technique, and the privacy-preserving distributed selective stochastic gradient descent (DSSGD).

#### Nonprotective Training

**Figure 2:**
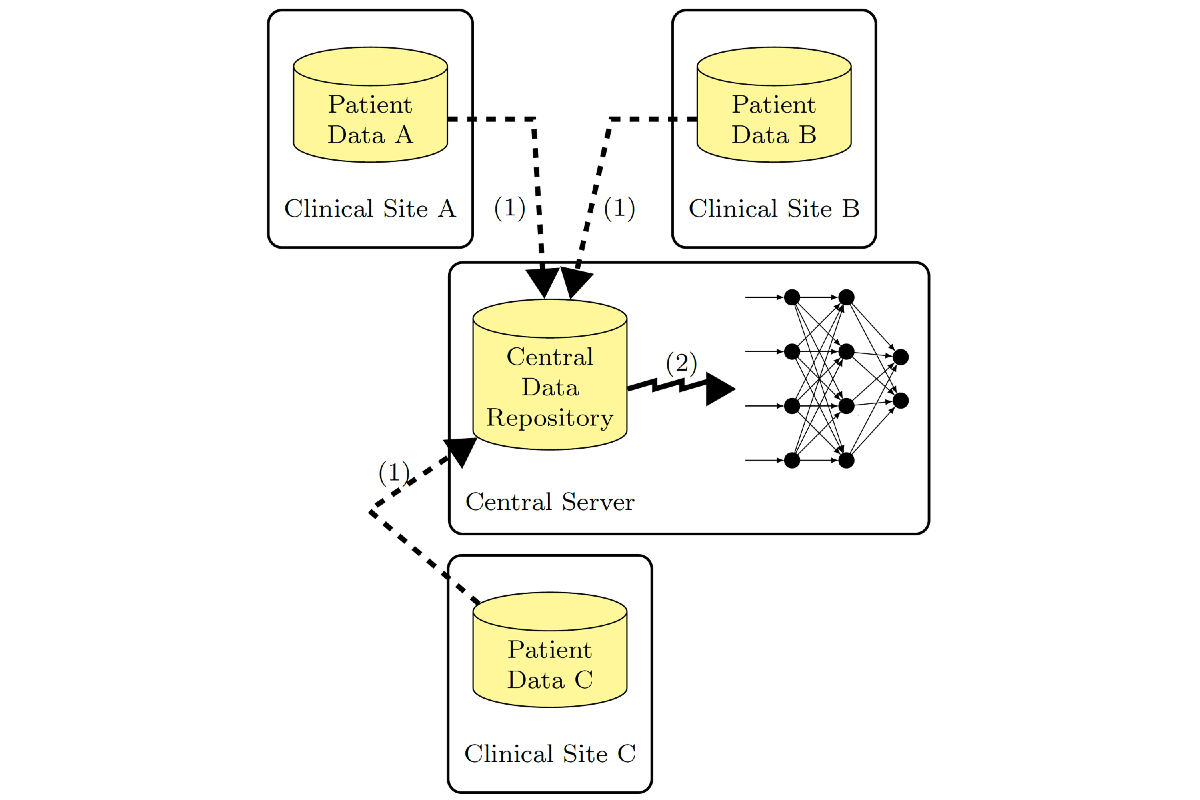
Nonprotected training with shared data.

The nonconfidential training (Figure 2) relies on central data processing. The dashed arrows denote the disclosure of the local training data, which leads to a large central data set. Usually only one entity controls the training by distributing tasks and data to workers of a large computing cluster [18]. The jagged arrow marks the distributed training supervised by the central server. The server has full control over all data and over the training procedure. Hence, this method is not suitable for protecting local data but enables fast training with full information integration.

#### Semiprotective Round Robin Training

**Figure 3:**
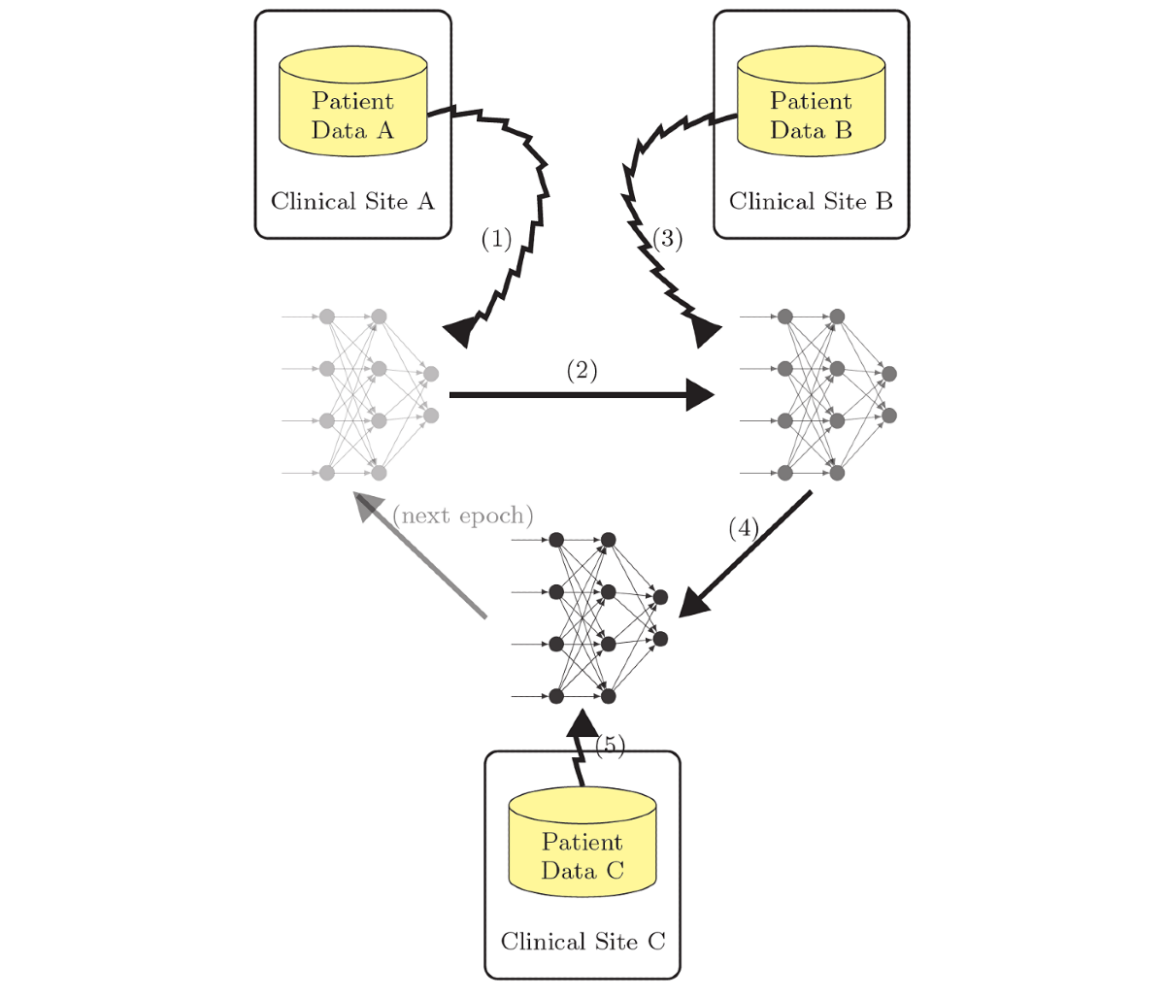
Semiprotective round robin training with local data.

An initial step toward privacy protecting training is to keep local training data secret and just share the NN that is trained by applying a round robin algorithm (Figure 3). In a first step, worker A trains the network using only its local data (jagged arrow [1]). After it is finished, the trained network is handed to the second worker (solid arrow [2]) who improves the network further by training with its local data (jagged arrow [3]). The other workers contribute analogously until the first training epoch is finished. Afterward the next round can be conducted in the same way. This training is similar to the cyclical weight transfer presented by Chang et al [13].

Although this protocol allows the workers to initially keep the local data secret, it does not prevent leakage of personal information through the passing on of the trained network. The parameters might still reveal parts of confidential information that have been used for training.

#### Distributed Selective Stochastic Gradient Descent

**Figure 4:**
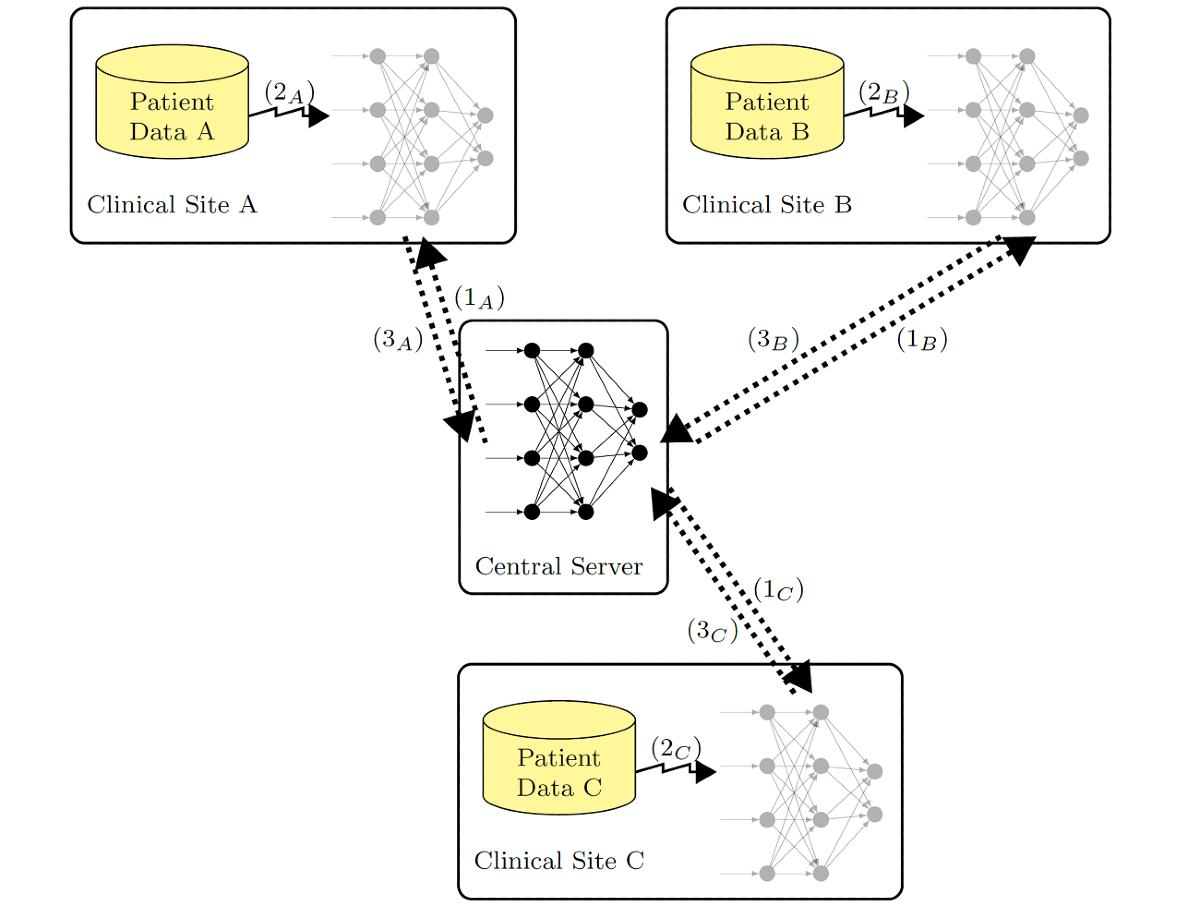
Distributed selective stochastic gradient descent with local data.

DSSGD enables an exclusively local training and additionally allows every participant to decide how much information about local parameters is shared (Figure 4). The following description of DSSGD solely corresponds to the version used for our experiments. We would like to point out that there are several other options suggested by Shokri and Shmatikov [4].

The basic idea of gradient descent in general is to find a (local) minimum of the loss function by adapting the weights along their negative gradients. In the fully stochastic version, every gradient is computed over only one sample at a time. Hereafter, the phrase stochastic gradient descent refers to this kind of gradient computation.

Let **p** be the flattened vector representing all parameters of the network (ie, *W_i_*, *W_c_*, *W_o_*, **b_i_**, **b_c_**, **b_o_** of both LSTM types and *W_1_*, *W_2_*, **b_1_**, **b_2_** of the feedforward layers). The cross-entropy error of the weights with respect to one training text *T* is defined as

*E*(**p**) = –∑_i=1,…,n_ (class(**t_i_**)⋅ln(**a_i_**))

where class(**t**_i_) ∈{0,1}^29^ is the one-hot representation of the correct class of token **t_i_**.

After the error has been computed in the forward pass, the gradient of *E*(**p**) can be determined in the backward pass using backpropagation. The individual parameters are then updated in the following way:

**p**_j_ = **p**_j_ – η ⋅ ∂E/∂**p**_j_ (**p**)

where η denotes a fixed learning rate.

The peculiarity of DSSGD is that the learning is conducted in a collaborative fashion but without a central manager (Figure 4). Every participating worker computes error gradients by using only its local data (jagged arrows). A certain number of local partial derivatives is shared with the other workers (dotted arrows) via the central server after every epoch. This fraction of shared derivatives θ can be set by local data curators. In order to prevent unwanted divulgation of private information, the absolute values of shared scalars can be clipped to an individually defined minimum/maximum γ. Moreover, some lower bound τ can be chosen to omit sharing updates with information that is too small. All those additional settings are unknown to other workers.

The central server is solely used to upload and download subsets of local derivatives asynchronously. It does not manage the local workers like a central manager and does not have access to the local training data.

In the following text, the local training as well as the parameter exchange is described in more detail. In the beginning, different parties agree upon a topology of the network and a learning rate. On this basis, the global weights **p_glo_** are initialized at the server. Beside the single set of weights, the server maintains a statistic for every weight indicating how often it has been updated by workers.

**Figure 5:**
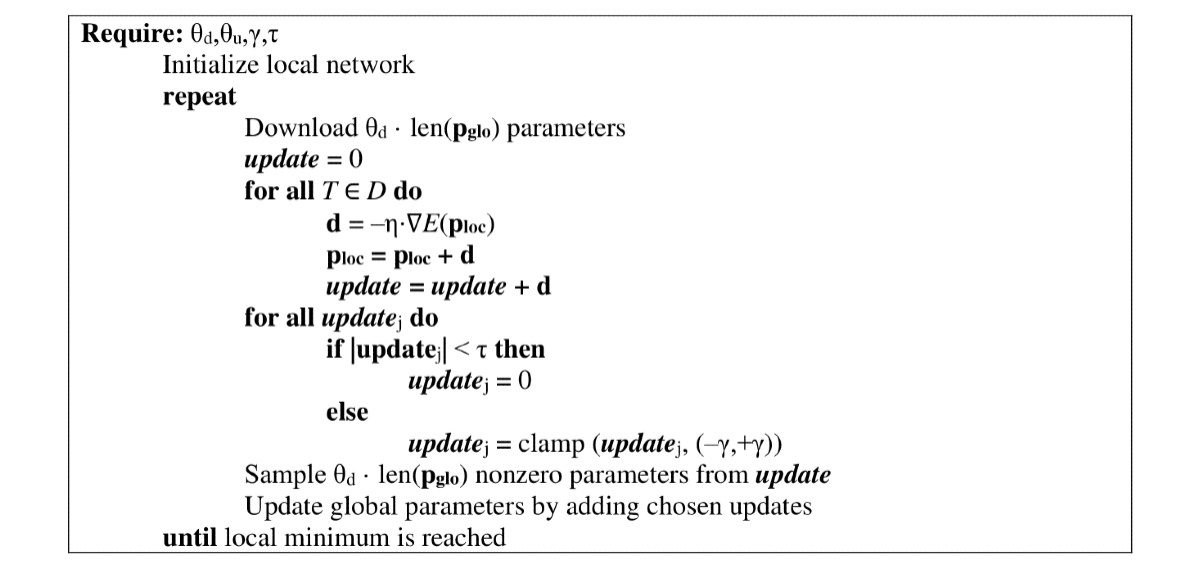
Local training procedure [4].

Figure 5 summarizes the work conducted by one single worker where *D* denotes the local training set. In the beginning, a worker builds a local copy of the network collectively agreed upon. Additionally, the local learning rate is set to the globally fixed value. A single epoch of the local training begins with the download of a subset of the global weights. The size of this set is specified by the local hyperparameter θ_d_. The θ_d_ ⋅ len(**p_glo_**) parameters with the highest global update statistics are chosen for the download. By adjusting θ_d_, the worker can decide how much other workers can influence its final result. In the subsequent step, simple stochastic gradient descent is used to improve local weights. After one epoch, the weight updates that are sent to the server are chosen. For this purpose, the first step is to check for every update whether its absolute value exceeds the threshold τ determined in the beginning. If this is not the case, this single update value is not communicated. Second, the remaining updates are clamped to the interval [–γ,+γ]. The third and last part of the selection process is based on randomness. To meet the upload rate θ_u_, the set of possible updates is sampled uniformly at random. The training is ended when a minimum or any other stopping criterion is reached. A comprehensive assessment of the passive protection assured by the presented method can be found in the original paper [4].

### Experiments

To evaluate the performance of the network for deidentification and the different training methods, we used a data set also used by Dernoncourt et al [3] for training the noncollaborative version. The basis of this data set is 1304 real longitudinal patient records for 296 diabetic patients provided by Partners Healthcare and adapted by Stubbs and Uzuner [15] for the 2014 i2b2 deidentification challenge. These notes were collected at different hospitals and are written in natural language (English) by caregivers to document or communicate patients’ medical history, physical and mental state, prescriptions, and other events. During the generation of this gold standard set, several human annotators marked critical PHI in the notes, which was later replaced by realistic surrogate data. Names, for example, have been replaced by imaginary names. See Stubbs and Uzuner [15] for a detailed description of the data set and the replacement methods.

The full training subset contains around 598,000 tokens with approximately 17,000 instances of PHI, while the subset used for testing consists of approximately 387,000 tokens including more than 11,000 instances of PHI. Records of patients represented in the training set are not contained in the test set. Due to the class imbalance, the F1 measure was used to quantify performances during experiments.

For the tokenization of the data sets, we made use of the tokenize package published as part of the Natural Language Toolkit. The Word2Vec embedding was generated with the help of the Gensim library and was trained on all English Wikipedia articles available in the beginning of November 2018. All networks were implemented with the help of the open source software library TensorFlow. We used graphics processing unit support based on the CUDA platform to accelerate training.

The first experiment (A) was conducted to get baseline results. We trained one RNN as described in section Recurrent Neural Network for deidentification using the full training data set. This training corresponds to the nonprotective case outlined in section Nonprotective Training. Plain stochastic gradient descent with a learning rate of 0.9 was used for the optimization. After every epoch, the performance was determined with respect to the test set. The hidden states, the cell states, and the biases of LSTMs used in the token embedding layer were of size 128. Thus, the weight matrices were in space 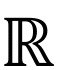
^128×256^ and **e_i_** ∈ 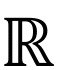
^556^. During training, dropout with probability 0.5 was applied to the sequence (**e_1_**,..., **e_n_**) to counteract overfitting that might have been introduced by the large number of training epochs. The single LSTM of the label prediction layer held parameter vectors that were all of size 100 and hence output the sequence (**d_1_**,..., **d_n_**) with d_i_ ∈ 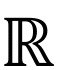
^200^. For the final two feedforward layers, the weights were *W_1_* ∈ 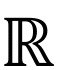
^100×200^ and *W_1_* ∈ 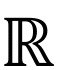
^29×100^.

Experiment B was performed according to the description in section Semiprotective Round Robin Training. The full training set was subdivided into 5 disjoint private sets of similar size. All records of one patient were kept in the same subset. The training was performed in a round robin fashion using stochastic gradient descent with a learning rate of 0.9 at each worker. The test set was, as in all experiments, left untouched and tested against after every full epoch (ie, after 5 local epochs).

In a third experiment (C_0.1), the collaborative privacy-preserving training (Distributed Selective Stochastic Gradient Descent section) was tested. For this purpose, the global network topology was chosen, as in the previous experiments. We trained 5 RNNs collaboratively that asynchronously shared some of their weight updates. The training data were distributed as in experiment B. In contrast to the previous experiment, the nets were tested against the global test set after every local epoch, since there is no global epoch in this setup.

Thus, there are 5 results for every epoch. The training hyperparameters for all nets were θ_d_=0.1, θ_u_=0.5, γ=10, τ=0.0001, while the global learning rate of DSSGD was set to 0.9. Afterward we conducted a similar experiment with the only difference being that θ_d_ was set to 0.5 (experiment C_0.5).

Experiment D was run in the same way as experiment C_0.1 except for the fact that this time the 5 networks did not communicate at all and just trained on their local sets. Again, we made use of simple stochastic gradient descent with a learning rate of 0.9. A summary of all experiments can be found in Table 2.

**Table 2:** Summary of the experiments.

Name	Learning strategy
A	Centralized training using stochastic gradient descent (learning rate: 0*.*9)
B	Collaborative training of 5 workers using the round robin method (learning rate: 0*.*9)
C_0*.*1	Collaborative privacy preserving training of 5 workers with DSSGD^a^ (θ_d_ = 0.1, θ_u_ = 0.5, γ = 10, τ = 0.0001; learning rate: 0.9)
C_0*.*5	Collaborative privacy preserving training of 5 workers with DSSGD (θ_d_ = 0.5, θ_u_ = 0.5, γ = 10, τ = 0.0001; learning rate: 0.9)
D	Local training without collaboration of 5 workers using stochastic gradient descent

^a^DSSGD: distributed selective stochastic gradient descent.

## Results

To obtain results that can be compared to the outcomes achieved by Dernoncourt et al [3], we adopted their scoring. Thus, every token labeled as PHI by the network was considered a true positive result if and only if this token was actually some PHI. The scoring, therefore, does not consider the PHI classes but only the decision PHI versus non-PHI.

**Figure 6:**
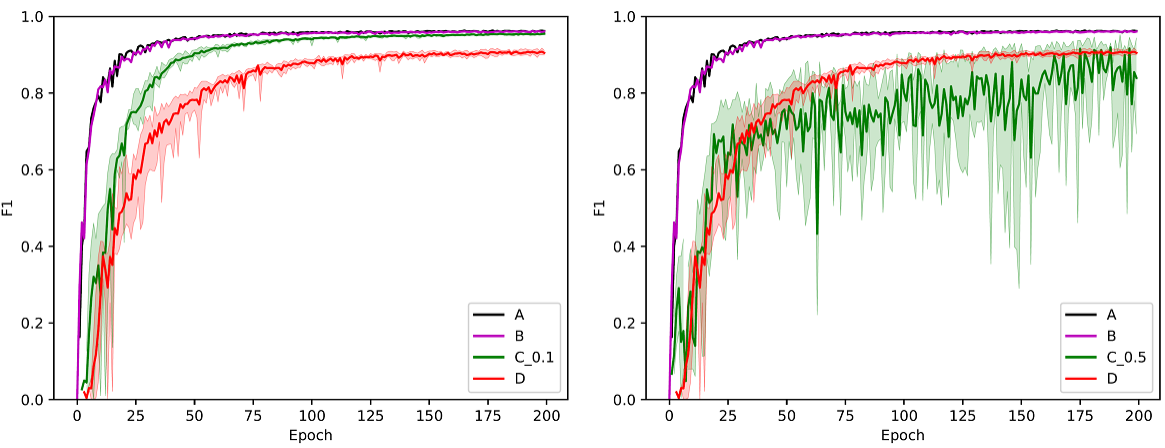
Performances achieved on the test set during the experiments including C_0.1 (left) or C_0.5 (right).

In all experiments the nets underwent 200 epochs of training. The illustration on the left in Figure 6 depicts the development of F1 scores achieved on the test set. The black line corresponds to experiment A and the magenta one to experiment B. Note that both lines are very similar and partially overlap. For the other experiments, there are 5 results per time point. The solid green line depicts the mean F1 values achieved in experiment C_0.1, while the area colored in green indicates the full range in which the values fell. Similarly, the results of experiment D are represented. For these measurements, the color red was used. For reasons of clarity, the results of experiment C_0.5 are depicted in the image on the right. Again, these results are shown in green. The remaining lines are the same as in the illustration on the left. The final scores are given in Table 3.

**Table 3:** Scores after 200 training epochs.

Experiment	F1		
	Minimum	Mean	Maximum
A	—	0.962	—
B	—	0.961	—
C_0.1	0.955	0.955	0.956
C_0.5	0.694	0.840	0.944
D	0.900	0.905	0.911

## Discussion

Using real-world clinical data, our study shows that collaborative privacy-preserving training enables a NN-based detection of PHI in clinical free-text records. The approach, thus, solves the dilemma of requiring machine learning techniques for easily adapting a PHI detection system to heterogeneous clinical documentation while avoiding disclosure of locally stored patient data.

Comparisons of the results achieved by the networks trained with plain stochastic gradient descent (A and D) underline the intuitive assumption that larger training data sets lead to better performances.

The similarity between the results of experiments A and B originates from the fact that both training methods are equivalent except for the kind of shuffling of samples during training. If the only aim is to keep the exact local data private and not to protect as much private information as possible, the round robin technique (or cyclic weight transfer) is the best choice. If, however, a real privacy-preserving collaborative learning mechanism is need as in the presented medical domain, DSSGD is the correct training algorithm. It offers both privacy preservation and good performance. The results of experiments C_0.1 and D show that a lack of sufficiently large local training data sets can be compensated by applying collaborative privacy-preserving training based on DSSGD. This finding is in line with the observations made by Shokri and Shmatikov [4] and the results obtained by Liu et al [14] who tested the protocol with some smaller nonrecurrent neural networks. By applying the collaborative training, all workers reach similar outcomes rapidly. After 25 epochs, all nets perform better than the ones trained with the corresponding noncollaborative version (D). The local F1 values all tend to the results achieved in the centralized version (A). During all experiments, a rather high learning rate was used that might have led to nonoptimal solutions. However, since it was kept constant throughout all 5 experiments, the insights gained by comparison are still valid. Another shortcoming of our experiments is that the γ value was set without using a calibrating data set as is suggested by Shokri and Shmatikov [4].

During experiments C_0.1 and C_0.5, the importance of well-chosen communication parameters θ_u_, θ_d_ became apparent. If the download rate is set to high, the global results interfere too much with local training sessions. This leads to high fluctuations and prevents the local weights from converging.

## Abbreviations

DSSGD: distributed selective stochastic gradient descent
i2b2: Informatics for Integrating Biology and the Bedside
LSTM: long short-term memory
NN: neural network
PHI: protected health information
RNN: recurrent neural network
